# Identification of an unannotated early embryonic *single-minded* transcript in the yellow fever mosquito *Aedes aegypti*

**DOI:** 10.17912/micropub.biology.002236

**Published:** 2026-08-26

**Authors:** William Reid, Marc S. Halfon

**Affiliations:** 1 Department of Biochemistry, University at Buffalo-State University of New York, Buffalo, New York, United States

## Abstract

The yellow fever mosquito, *Aedes aegypti* , is a cosmopolitan species that serves as the vector of multiple disease causing agents including dengue, chikungunya, Zika, and yellow fever viruses. The genome of *Ae. aegypti* has been characterized at the chromosome level, but further manual refinement is required for genes and isoforms with transient expression or low abundance. Here we report on the identification of an early embryonic transcript for the *single-minded (sim* ) *gene* in *Ae. aegypti* , and present the putative promoter for the transcript. The identification of an early-driven transcript is consistent with the annotation for *sim* in *Drosophila melanogaster.*

**Figure 1. The (revised) sim locus of Ae. aegypti:** (A) Conservation of the *sim* early transcript promoter region for five species of *Aedes* spp. The upper purple histogram represents the percentage of conservation for all five species; nucleic acid sequences are color-indicated below. (B) Genomic position of the three isoforms of *sim* including the previously un-annotated early promoter. (C) Time-course RT-PCR of the late, ovarian, and early transcripts for the *single-minded* ( *sim* ) gene in *Aedes aegypti (AAEL011013).* The early transcript is present from 4h AEL and sharply decreases in expression by 18h AEL, while the late ( *RB* ) transcript is present from 10h AEL and remains the dominant transcript throughout embryogenesis and the other life stages tested, with the exception of the male carcass, where it is absent, and the ovary. The ovarian ( *RC* ) transcript is detected uniquely in the ovaries, where it is the dominant transcript. L1-L4: 1st instar larva through 4th instar larva; mP: male pupae; fP: female pupae; mH: adult male heads; mC: adult male carcass without heads; fH: adult female heads; fC: adult female carcass without heads; OV: 48h post blood meal ovaries.

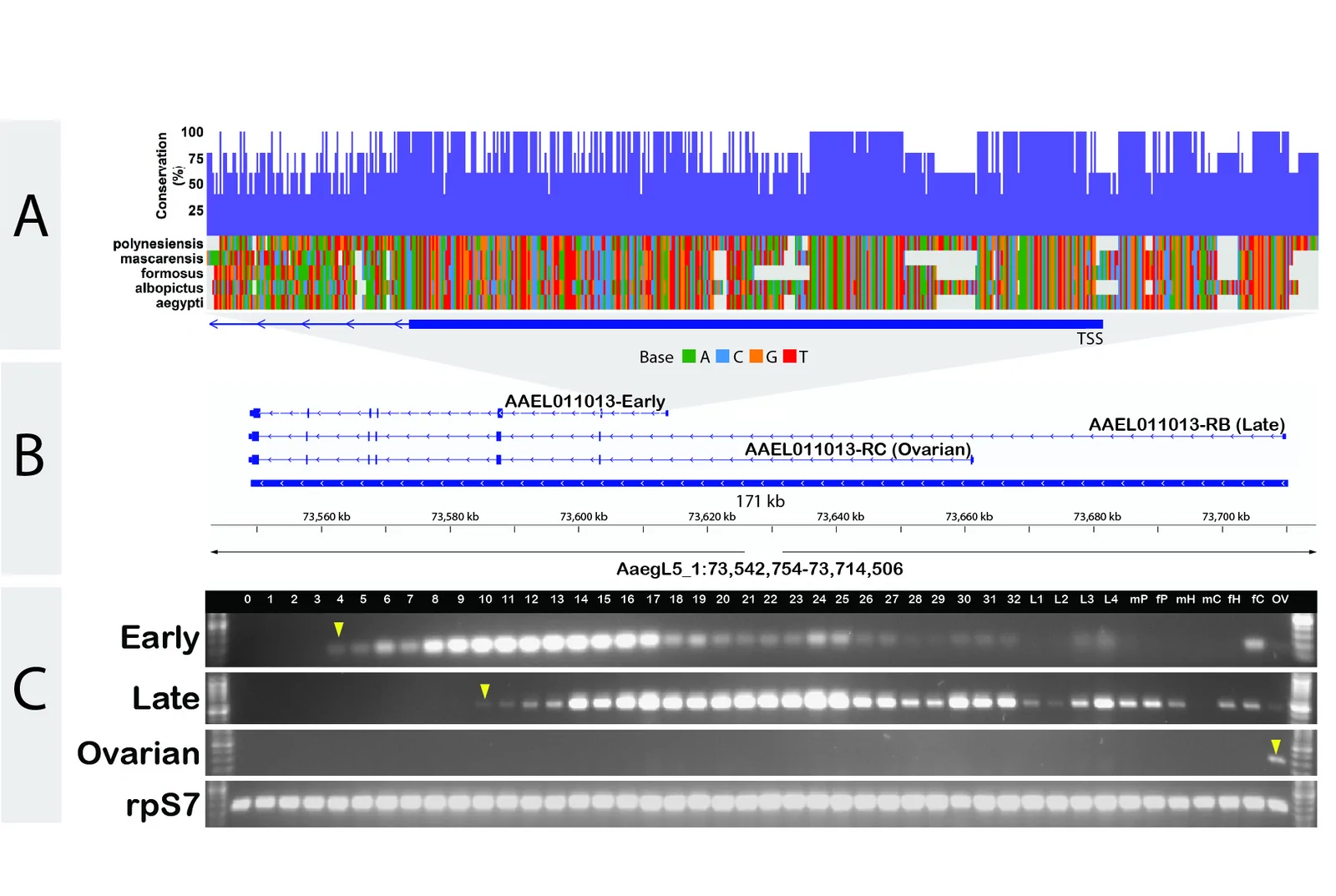



## Description

Accurate characterization of gene structure and transcript isoform expression is an essential prerequisite for studying a gene’s function and regulation. During insect embryogenesis, the *single-minded (sim* ; FlyBaseID:FBgn0004666 *)* gene is a critical and conserved regulator of the central nervous system midline (Nambu et al., 1991; Zinzen et al., 2006; Linne et al., 2012). Sim, a bHLH-Pas domain transcription factor, heterodimerizes with the related bHLH-Pas protein Tango to bind to regulatory elements and stimulate the expression of key genes involved in the development of midline neuronal and glial cell lineages (Nambu et al., 1990; Wharton et al., 1994; Sonnenfeld et al., 1997; Watson and Crews, 2012). *sim* ’s structure, transcriptional profile, and regulation are all well-characterized in *Drosophila melanogaster* , where *sim* is expressed from the early blastoderm stage and remains expressed throughout embryogenesis (Thomas et al., 1988; Wheeler et al., 2006). Post-embryogenesis, *sim* is involved in axon guidance in the brain, development of the optic lobe and genital discs, and high expression in the follicular cells of the ovaries (Pielage et al., 2002; Freer et al., 2011; Oramas et al., 2023). Three promoters control the expression of *D. melanogaster sim* , giving rise to virtually-identical isoforms: an ‘early’ transcript, which is the predominant transcript from embryonic stages 5 through 9, a ‘late’ transcript, which is the predominantly expressed transcript from stage 9 onward throughout embryogenesis and in other developmental stages, and an ovarian isoform, which is expressed in the follicular cells of the developing egg, promoting maturation (Freer et al., 2011; Knapp et al., 2020). The ‘late’ embryonic promoter also produces a second nearly-identical alternatively-spliced transcript from roughly stage 9 throughout embryogenesis (Freer et al., 2011).

Recently, we have been studying how *sim* is regulated in the distantly-related mosquito *Aedes aegypti* (Schember et al., 2024; Sterling-Lentsch and Halfon, 2024). However, only two transcripts were annotated in the *Ae. aegypti* genome for the *sim* ortholog *AAEL011013* , leading us to wonder if the genome annotation was complete. Although RNA-seq data exist for *Ae. aegypti* embryos (e.g. Biedler et al., 2012; Akbari et al., 2013), an early transcript that is expressed transiently and only in the small population of midline cells could easily be missed in RNA-Seq characterization due to low coverage depth. Here, we report on the identification of such an early *sim* transcript, thus demonstrating equivalency in gene structure and promoter usage between *D. melanogaster* and *Ae. aegypti* .

To test for the presence of a distinct unannotated early *Ae. aegypti sim* transcript, we collected *Ae. aegypti* embryos at 4h after egg laying (AEL) and processed them for 5’-RACE. We identified a novel transcript with a transcription start site (TSS) 10,263 bp upstream of Exon II of *AAEL011013* ( Fig. 1B ) and confirmed its existence with allele-specific intron-spanning RT-PCR primers ( Fig. 1C ). This places the early *sim* promoter as the most proximal to the shared Exon II, with the *AAEL011013-RC* (ovarian) and *AAEL011013-RB* (late) TSSs being 58,005 and 106,578 bp upstream, respectively. These results are consistent with the *sim* gene structure of *D. melanogaster* , where the early promoter is the most proximal to the common Exon II of the three defined *sim* promoters. Further testing of the expression of all *sim* transcripts using allele-specific intron-spanning RT-PCR ( Fig. 1C ) demonstrated that the early *sim* promoter is active as early as 4h AEL and sharply decreases expression by 18h AEL, at which point embryos are approximately equivalent to *Drosophila* stage 10-12. Meanwhile, the late *sim* transcript ( *AAEL11013-RB* ) is first detectable at 10h AEL (stage 8-10) and continues to be expressed throughout embryogenesis and through all life stages with the exception of the male carcass. The third *sim* isoform, *AAEL11013-RC* , was detectable only in 48h post-bloodmeal ovaries. The onset of transcript expression in *Ae. aegypti* is therefore consistent with that in *D. melanogaster,* where expression from the early promoter tails off by stage 11 and the ovarian promoter drives expression exclusively in the ovaries (Freer et al., 2011). Detection of the early-promoter and *AAEL11013-RB sim* transcripts in female, but not male, carcass may be due to the presence of expression in ovarian tissue, since the assayed carcasses consisted of the entirety of the mosquito minus the head. Three in-frame ATG putative start codons are present within the early *sim* transcript and are conserved among four additional *Aedes* spp: *Ae. albopictus* , *Ae. formosus* , *Ae. mascarensis* , and *Ae. polynesiensis* ( Fig. 1A ). Alignment and conservation analysis of all five *Aedes* spp. shows two strong blocks of conservation at the beginning of the predicted TSS through the first 100 bp of exon I. A third block of conservation was identified spanning the predicted splice donor site ( Fig. 1A ), with conservation in the region between the promoter and the splice donor remaining ≥60%. Conceptual translation of the orthologous exon I in all *Ae* . spp. indicates that either of the two ATG present within exon I could be the start codon, encoding 53 or 61 predicted N-terminal residues respectively, as all species retain in-frame ORFs irrespective of start codon choice. As such, whether the putative exon I of the early *sim* transcript is entirely or only partially 5’UTR cannot be determined from our study. Local alignment of the *D. melanogaster* early *-* specific N-terminal residues against those predicted for the *Ae. aegypti* early-specific isoform suggests a moderate degree of conservation (28.6% identity; 42.9% similarity) *,* but whether or not these residues are translated or relevant for early Sim function remains to be determined *.* Our work further refines the structure of the *sim* locus in *Ae. aeygpti,* which will facilitate future studies, and underscores the need for careful manual annotation and experiments beyond bulk RNA-seq to ensure reliable description of minor transcripts *.*

## Methods

*Mosquito rearing. Ae. aegypti* (strain Liverpool) were maintained at 27°C and 75% humidity under a 12:12 (L:D) photoperiod. Larval mosquitoes were fed *ad libitum* with a slurry of ground Tetramin fish flake (Tetra, Blacksburg, VA), and adults were provided with raisins as a sugar source and defibrinated sheep blood (Hardy Diagnostics, Santa Maria, CA) supplemented with 1 µM ATP for blood feeding.

*Embryo collection and aging* . Five day old adult females were provided with a bloodmeal, and four days following, 20-30 hypergravid females were collected and placed into a 50 mL conical tube containing Whatman #1 filter paper wetted with deionized water. Females were allowed to oviposit for 1h and embryos were subsequently aged on wetted Whatman #1 filter paper at 27°C for the indicated time periods of “hours after egg laying” hours AEL.

*RNA extraction, 5’RACE, RT-PCR.* Total RNA was collected by homogenizing ~10 mg of embryos in Trizol (ThermoFisher Scientific, Grand Island, NY) according to the manufacturer’s instructions. A total of 3 μg of RNA collected from 4h AEL embryos was used for the 5’-RACE, utilizing the 5’-RLM Generacer kit (ThermoFisher Scientific, Grand Island, NY) and first strand synthesis was conducted using oligo dT and Superscript III reverse transcriptase (ThermoFisher Scientific, Grand Island, NY). PCR products were amplified using *sim* -specific reverse primers and RACE PCR products were sequenced using nanopore sequencing (Plasmidsaurus, Louisville, KY). The cDNA templates for the time course RT-PCR were reverse transcribed in 20 μL reactions from 500 ng of total RNA using the RevertAid First Strand cDNA synthesis kit (ThermoFisher Scientific, Grand Island, NY) according to the manufacturer’s instructions. First strand cDNA was subsequently diluted 5-fold and 1 μL was used for each of four 15 μL RT-PCR reactions specific for: 1) intron-spanning *sim* early transcript, 2) intron-spanning *sim* late transcript, 3) intron-spanning *sim* ovarian transcript, and 4) rpS7 control. Five μL of each PCR product was then run out on a 1.5% agarose gel in 1xTBE and stained with ethidium bromide.

*Promoter alignment.* The full loci for *sim* for *Ae. aegypti* (AaegL5.0; GCA_002204515.1) *, Ae. albopictus* (AalbF5; GCA_035046485.1) *, Ae. formosus* (Aaf_Bf05_pri1.0; GCA_052575885.1) *, Ae. mascarensis* (Am_MascCH02_pri1.0; GCA_052575835.1) *, and Ae. polynesiensis* (Smith_Apoly_02182021; GCA_051529985.1) were downloaded from NCBI (ncbi.nlm.nih.gov) and aligned using MAFFT. The DNA regions matching to the predicted early *sim* transcript promoter from *Ae. aegypti* were then manually extracted and aligned to identify regions of full and partial conservation among the five mosquito species.

## Reagents

**Table:** 

**Strain**	**Genotype**	**Available from**
Liverpool	*Aedes aegypti*	BEI reagents
		
**Kit**	**Source**	**Description**
GeneRacer kit with SuperScript III RT	ThermoFisher Scientific	RNA ligase mediated 5’ adapter tagging for RACE reaction
RevertAid First Strand cDNA synthesis kit	ThermoFisher Scientific	Reverse transcriptase for first strand cDNA synthesis from time course samples
HotStar Taq	Qiagen	Taq polymerase for RT-PCR amplification
Sheep blood	Hardy Diagnostics	Blood source for adult female mosquito feeding
Tetramin fish flakes	Tetra	Food source for larval mosquitoes
		
**Primers**	**5’-3’**	
Aegsim_EIearly-F1	ATGACACCGCTGATGGACTG	
Aegsim_EIearly-II-R	CGTGGCAAGACCCAATATATTC	
Aegsim_EIlate-F1	AAGTTTGCCAAAGTCGTCGT	
Aegsim_EIlate-II-R	GCGTGGCAAGACCTTAGAATTTG	
Aegsim_E1ovary-F2	AGGTGGTCAGTTTGTTGCAGA	
Aegsim_E1ovary-II-R	GCGTGGCAAGACTTTTATAAGAC	
rpS7-F	GGAGAAGAAGTTCTCCGGCAAG	
rpS7-R	TGAAGGTGTCGACCTTGTGTTC	
